# Survival of Normal and Chlorine-Stressed Pathogenic and Non-Pathogenic *Vibrio parahaemolyticus* Under Adverse Conditions

**DOI:** 10.5812/jjm.9313

**Published:** 2014-03-01

**Authors:** Mehdi Zarei, Mohammad Hadi Eskandari, Somayeh Keshtkaran

**Affiliations:** 1 Department of Food Hygiene, Faculty of Veterinary Medicine, Shahid Chamran University of Ahvaz, Ahvaz, IR Iran; 2 Department of Food Science and Technology, College of Agriculture, Shiraz University, Shiraz, IR Iran

**Keywords:** *Vibrio parahaemolyticus*, Chlorine, Survival, Stress

## Abstract

**Background:**

*Vibrio parahaemolyticus* is an important human pathogen which can cause gastroenteritis when consumed in raw or partially-cooked seafood. The pathogenesis of *V. parahaemolyticus* is based on the presence of virulence factors: the thermostable direct hemolysin (TDH) and TDH-related hemolysin (TRH), encoded by the *tdh* and *trh* genes, respectively.

**Objectives:**

The present study aimed to evaluate the survival of normal and chlorine-stressed cells of pathogenic and non-pathogenic *V. parahaemolyticus* under adverse conditions.

**Materials and Methods:**

Normal and chlorine-stressed cells of pathogenic and non-pathogenic *V. parahaemolyticus* were subjected to environmental stresses such as low storage temperature (4°C and -18°C), high incubation temperature (50°C) and high NaCl content (20%). Viable counts were then made at various time intervals by surface plating on TSA-2.0% NaCl, and the survival rates of the cells were determined and compared.

**Results:**

Findings of the current study revealed that the normal cells of pathogenic and non-pathogenic *V. parahaemolyticus,* as well as the chlorine-stressed cells of both strains behave similarly under adverse conditions. In addition, chlorine stress increased the susceptibility of pathogenic and non-pathogenic *V. parahaemolyticus* to incubation at 4°C, and the presence of high NaCl content in the medium. However, chlorine stress did not significantly affect the thermal tolerance of pathogenic and non-pathogenic *V. parahaemolyticus*, and the susceptibility to incubation at -18°C.

**Conclusions:**

Chlorine-stressed cells of *V. parahaemolyticus* were more susceptible to adverse conditions than the non-stressed ones. Pathogenic and non-pathogenic strains showed the same survival characteristics under the adverse conditions. These results should be considered in the development of food preservation measures.

## 1. Background

*Vibrio parahaemolyticus* is a Gram negative, halophilic bacterium recognized as an important food-borne pathogen worldwide. Consumption of raw or undercooked seafood, particularly shellfish, contaminated with *V. parahaemolyticus* may lead to development of acute gastroenteritis characterized by diarrhea, headache, vomiting, nausea, abdominal cramps and low fever (1, 2). Although the mechanism by which the organism infects humans has yet to be comprehensively determined, thermostable direct hemolysin (TDH) and TDH-related hemolysin (TRH) have been recognized as primary virulence factors in *V. parahaemolyticus*. The TDH and TRH hemolysins are produced by *V. parahaemolyticus* strains that respectively harbour *tdh* and *trh* genes. It is commonly admitted that TDH is almost exclusively associated with clinical isolates, with less than 5% of environmental isolates producing TDH. As described with TDH and its gene, the frequency of trh-positive *V. parahaemolyticus* strains in the environment appears to be very low (3-5).

 While preparing and processing food, microorganisms are commonly subjected to various stresses such as sanitizers, cold, heat, acid and preservatives. These stresses cause the injury or death of microorganisms and therefore are considered to hinder their proliferation resulting in longer and safer food preservation (6-8). Chlorine is the most widely used agent for disinfecting water which is usually added to water in the gaseous form, calcium, or sodium hypochlorite. Chlorination of the washing water is frequently used to reduce or remove microorganisms from the fishing vessels, surface of utensils, and equipment thus promoting a hygienic environment in food processing operations. Although chlorination is performed in order to kill the organisms, it is not always completely effective. Some organisms will only be injured by the chlorine and some will completely survive after the treatment. The process efficacy is influenced by dose, contact time, pH and presence of organic compounds (9).

We recently reported the seasonal prevalence of *V. parahaemolyticus*, including TDH-positive strains in shrimp samples in the South-western part of Iran (10). Since harvested shrimp in Iran, before packaging or processing, are washed with 2-7ppm chlorine containing water (11), *V. parahaemolyticus* is commonly exposed to chlorine. This exposure may induce some changes in the growth and survival characteristics of the remaining *V. parahaemolyticus* cells, regardless of the pathogenicity.

## 2. Objectives

The present study aimed to:

(i) compare the survival of pathogenic and non-pathogenic *V. parahaemolyticus* under adverse conditions,
(ii) investigate the effect of chlorine stress on the susceptibility of pathogenic and non-pathogenic *V. parahaemolyticus* to other environmental stresses, and
(iii) compare the behavior of the chlorine-stressed cells of pathogenic and non-pathogenic *V. parahaemolyticus* under adverse conditions.

## 3. Materials and Methods

### 3.1. Microorganisms

*V. parahaemolyticus* (*tdh*^-^, *trh*^-^, Prince of Songkla University-PSU 2591) and *V. parahaemolyticus* (*tdh*^+^, *trh*^-^, PSU 2551) were the organisms used in the current study. The test organisms were first activated by two successive transfers in Tryptic Soy Broth supplemented with 2% NaCl at 35°C for 18-20 h. This activated culture served as the inoculum.

### 3.2. Preparation of the Chlorine Solutions and the Chlorine-Stressed Cells

Chlorine solutions were made using calcium hypochlorite (Merck, Darmstadt, Germany) and sterile deionized water that had no chlorine demand. Total chlorine was determined according to ISO 7393-3: 1990, using iodometric titration method (12).

To prepare the chlorine-stressed cells of pathogenic and non-pathogenic *V. parahaemolyticus*, first, the effect of chlorine concentration on the survival of pathogenic and non-pathogenic *V. parahaemolyticus* was investigated. Inoculum cultures of pathogenic and non-pathogenic *V. parahaemolyticus* (1.0 mL) were inoculated into 50 mL of deionized water-2.0% NaCl (pH 7.5) containing 0.0, 1.75, 3.5, and 7.0 ppm chlorine at an initial population of 10^6^-10^7^ cfu/mL. They were all incubated at 35°C for a 5 h period. At different time intervals, the test organisms’ survival was determined. Based on these results (data not shown), 3.5 ppm chlorine was selected for preparation of the chlorine-stressed cells.

To achieve this goal, inoculum culture of *V. parahaemolyticus* (1.0 mL) was first harvested by centrifugation (10000 rpm, 5 min) and washed with sterile deionized water-2.0% NaCl (pH=7.5) twice. They were then resuspended in 10.0 mL of the same water containing 3.5 ppm chlorine and then held at room temperature for 30 min. The cell suspension served as the source of chlorine-stressed cells and was used in the experiments described in the present study. The control cells were also prepared by resuspension in sterile deionized water-2.0% NaCl (pH 7.5) at room temperature but they were not subjected to chlorine stress.

### 3.3. The Effect of Chlorine Stress on the Survival of Pathogenic and Non-Pathogenic V. parahaemolyticus Under Adverse Conditions

To determine the effect of chlorine stress on the survival of pathogenic and non-pathogenic *V. parahaemolyticus* at 4°C, 1.0 mL of the chlorine-stressed or control cells of pathogenic and non-pathogenic *V. parahaemolyticus* was inoculated into 50.0 mL of TSB-2.0% NaCl (precooled at 4°C) at an initial population of ca.10^6^ cfu/mL and incubated at 4°C for a 10 day period. Samples were taken at various time intervals to determine the cells’ viability (13, 14). To investigate survival at -18°C, 0.1 mL of the chlorine-stressed or control cells of pathogenic and non-pathogenic *V. parahaemolyticus* was inoculated into each of the culture tubes containing 5.0 mL of the precooled TSB-2.0% NaCl at an initial population of ca.10^6^ cfu/mL. Cells were then stored at -18°C in a freezer for 7 days. At various storage times, tubes were removed from the freezer and contents were thawed at room temperature The viability of the cells was then determined (13, 14).

To determine the thermal tolerance of the test organisms, 1.0 mL of the chlorine-stressed cells or the control cells of pathogenic and non-pathogenic *V. parahaemolyticus* was inoculated into 50.0 mL TSB-2.0% NaCl (pretempered at 50°C) at an initial population of ca.10^6^ cfu/mL, and submerged in a preheated water bath at 50°C for 30 min. Samples were taken at different time intervals, and the survival of the cells was subsequently determined (13, 14). To examine susceptibility to high NaCl content, 1.0 mL of the chlorine-stressed cells or the control cells of pathogenic and non-pathogenic *V. parahaemolyticus* was inoculated into 50.0 mL of TSB containing 20% NaCl at an initial population of ca.10^6^ cfu/mL. The cell suspensions were incubated at 35°C for a period of 6 h and the viability was determined after various durations of incubation (13, 14).

### 3.4. Enumeration of V. parahaemolyticus

To determine the viable population of *V. parahaemolyticus*, samples were first serially diluted with physiological saline solution. Viable counts were then made by surface plating 0.1 mL of the decimal diluted sample, each on duplicate plates of TSA-2.0% NaCl. Colonies were counted after 24 h of incubation at 35°C (13, 14).

### 3.5. Statistical Analysis

All experiments were performed in triplicate. SPSS software version 16 was employed to analyze the data through the Repeated Measure ANOVA and Independent Samples t test. The significance levels were expressed at a 95% confidence level (P ≤ 0.05).

## 4. Results

### 4.1. Survival of Pathogenic and Non-Pathogenic V. parahaemolyticus Under Adverse Conditions

**Figure 1.**
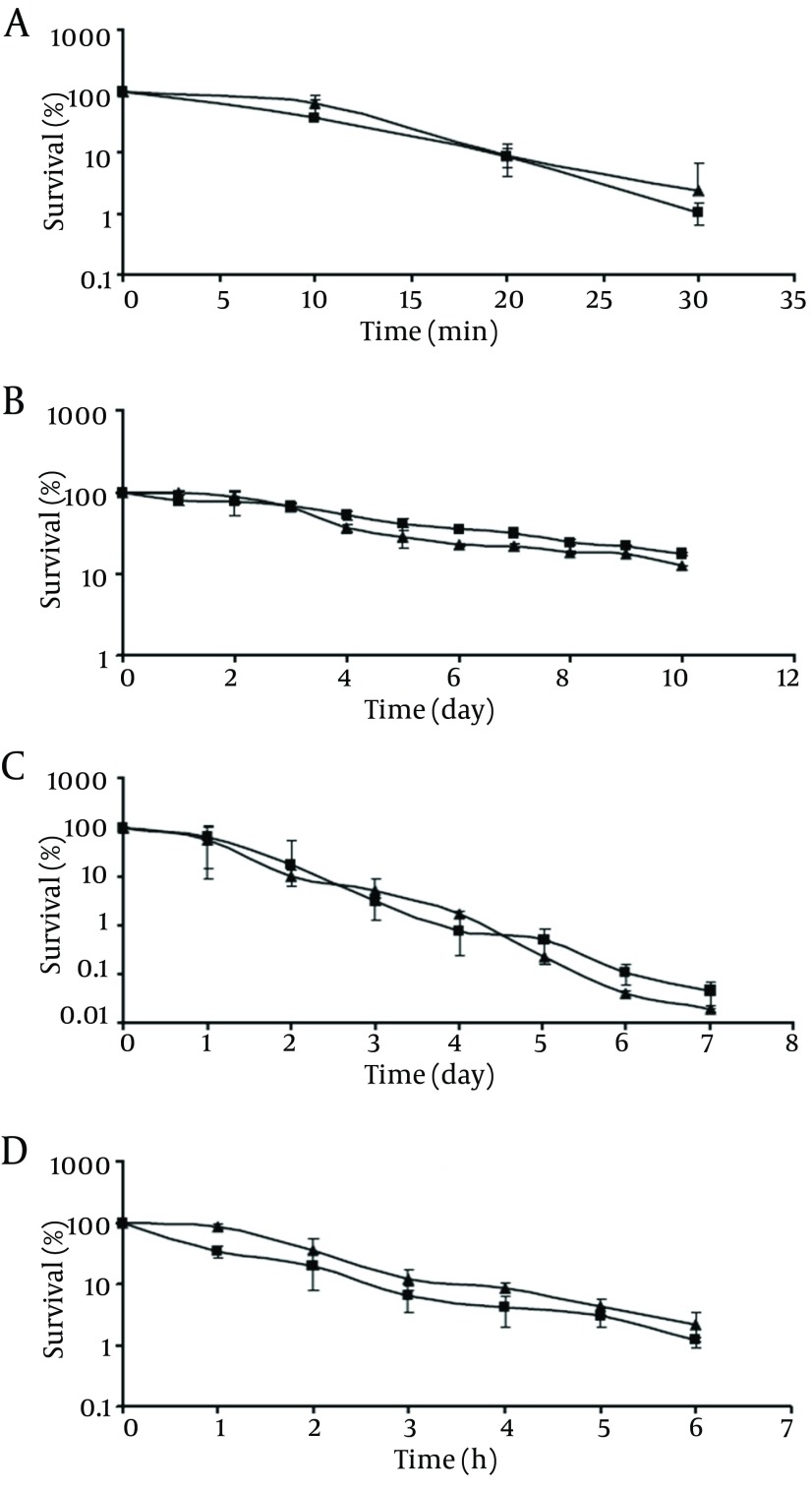
Survival of Pathogenic (■) and Non-Pathogenic (▲) *V. Parahaemolyticus* at 50°C (A), 4°C (B), -18°C (C) and in the Presence of 20% NaCl (D) The initial populations were 10^4^-10^5 ^cfu/mL. Survival rates were obtained by dividing the survival populations by the initial populations which corresponds to 100%.

As shown in Figure 1A-C, the survival rates of pathogenic and non-pathogenic *V. parahaemolyticus* decreased as the incubation time increased, in all the incubation temperatures tested. At the end of the incubation period at 50 °C (Figure 1A), pathogenic and non-pathogenic strains showed survival rates of ca. 1.1% and, 2.4%, respectively. When stored at low temperatures, viable populations of both strains showed a decreasing trend upon extending the storage time. At the end of the 10-day storage period at 4°C, pathogenic and non-pathogenic strains showed survival rates of ca. 17.3% and 12.5%, respectively (Figure 1B). *V. parahaemolyticus* cells, regardless of their pathogenicity, declined to a greater extent at -18°C (Figure 1C) rather than at 4°C. At the end of the 7-day storage period, pathogenic and non-pathogenic strains showed survival rates of ca. 0.04% and 0.02%, respectively (Figure 1C). However, at the end of the incubation period, no significant difference was observed in the viable population of pathogenic and non-pathogenic strains in all the temperatures tested (P > 0.05). The same result was observed in the presence of 20% NaCl (Figure 1D), where at the end of the incubation time viable population of pathogenic *V. parahaemolyticus* decreased to 1.23%, which did not differ significantly from that of the non-pathogenic strain (2.18%).

### 4.2. The Effect of Chlorine Stress on the Susceptibility of Pathogenic and Non-Pathogenic *V. parahaemolyticus* to Various Environmental Stresses

#### 4.2.1. The Effect of Chlorine Stress on the Survival at Low Temperatures

**Figure 2.**
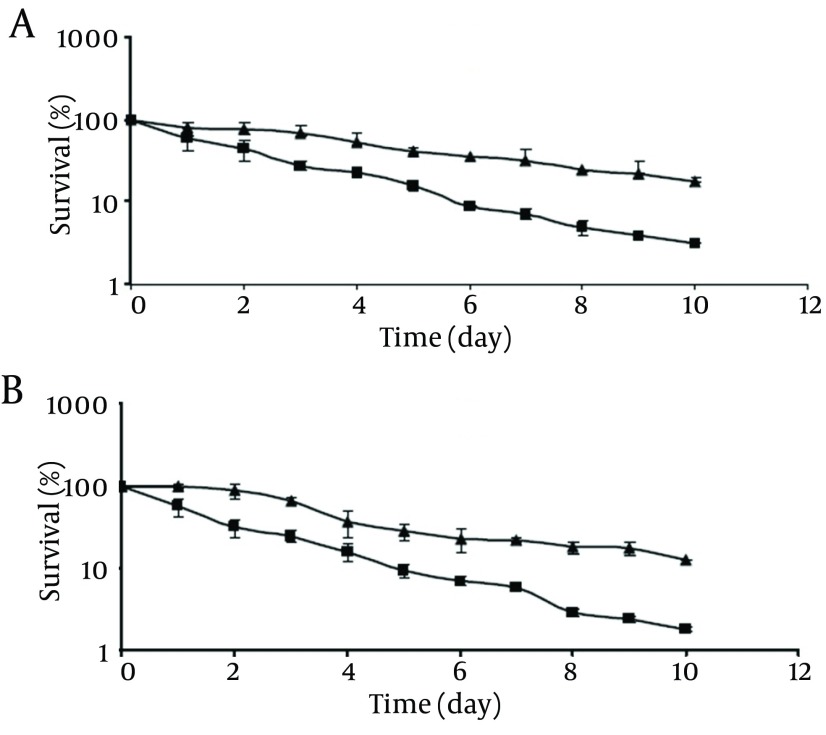
Survival of Pathogenic (A) and Non-Pathogenic (B) *V. Parahaemolyticus* at 4°C: Control Cells (▲); Chlorine-Stressed Cells (■) The initial populations were 10^4^-10^5^cfu/mL. Survival rates were obtained by dividing the survival populations by the initial populations which corresponds to 100%.

As shown in Figure 2, the survival rates of chlorine-stressed cells of pathogenic and non-pathogenic strains significantly decreased (P < 0.05) compared to those of the control cells as the storage time at 4°C was extended. At the end of the 10-day storage, the control cells of pathogenic and non-pathogenic strains showed survival rates of 17.4% and 12.6%, respectively. Meanwhile, chlorine-stressed cells of both strains exhibited a significantly (P <0.05) lower survival rates of 1.2% and 1.1%, respectively. Comparing the behavior of chlorine-stressed cells of pathogenic and non-pathogenic *V. parahaemolyticus* during storage at 4°C showed no significant difference (P > 0.05), similar to the normal cells.

**Figure 3.**
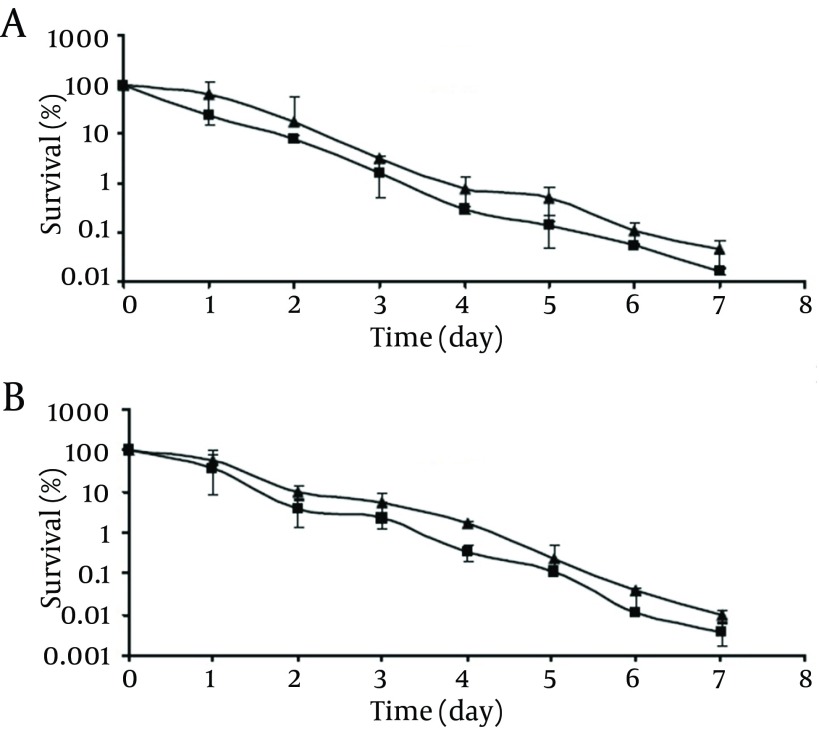
Survival of Pathogenic (A) and Non-Pathogenic (B) *V. Parahaemolyticus* at -18°C: Control Cells (▲); Chlorine-Stressed Cells (■) The initial populations were 10^4^-10^5^cfu/mL. Survival rates were obtained by dividing the survival populations by the initial populations which corresponds to 100%.

At -18°C, survival of both the control and chlorine-stressed cells of pathogenic and non-pathogenic strains showed a decreasing trend upon extending the storage time (Figure 3). Although during the storage period, viable populations of the control cells of pathogenic and non-pathogenic strains were higher than those of the chlorine-stressed cells, they did not differ significantly (P > 0.05). As observed at 4°C, no significant difference was observed in the survival of chlorine-stressed cells of pathogenic and non-pathogenic *V. parahaemolyticus* during storage at -18 °C (P > 0.05).

#### 4.2.2. The Effect of Chlorine Stress on the Thermal Tolerance

**Figure 4.**
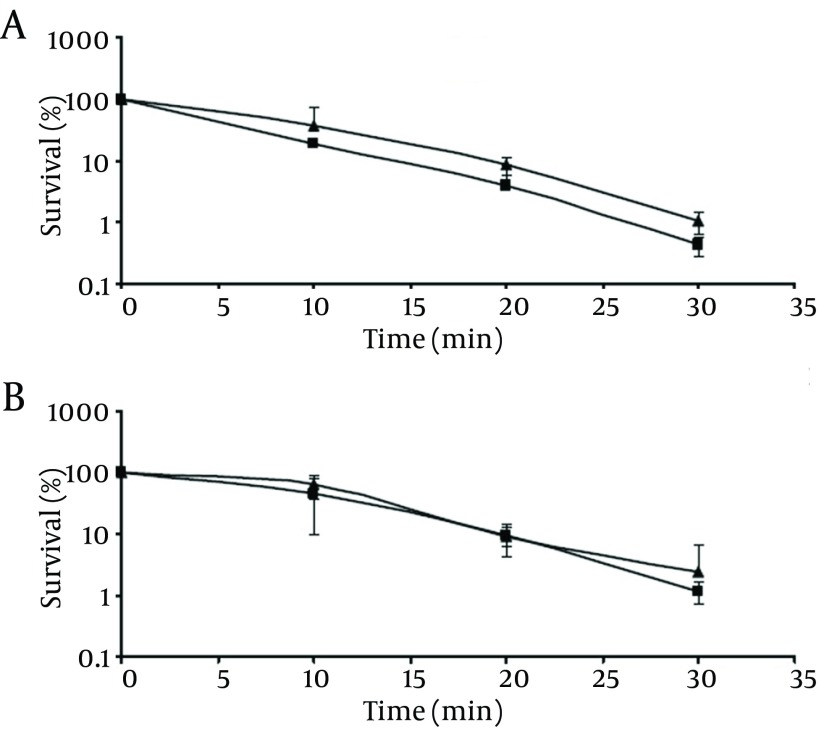
Survival of Pathogenic (A) and Non-Pathogenic (B) *V. Parahaemolyticus* at 50°C: Control Cells (▲); Chlorine-Stressed Cells (■). The Initial Populations Were 10^4^-10^5^cfu/mL Survival rates were obtained by dividing the survival populations by the initial populations which corresponds to 100%.

As shown in Figure 4, viability of both the control and chlorine-stressed cells, regardless of their pathogenicity, decreased as the incubation time at 50°C increased. At the end of the incubation period, control cells of pathogenic and non-pathogenic strains showed survival rates of ca. 1.1% and 2.4%, respectively, which did not differ significantly (P > 0.05) from those observed with chlorine-stressed cells of both strains (0.4% and 1.4% for pathogenic and non-pathogenic strains, respectively). Similar to the normal cells, comparing the survival of chlorine-stressed cells of pathogenic and non-pathogenic *V. parahaemolyticus* showed no significant difference (P > 0.05).

#### 4.2.3. Effect of Chlorine Stress on the Susceptibility to High NaCl Content 

**Figure 5.**
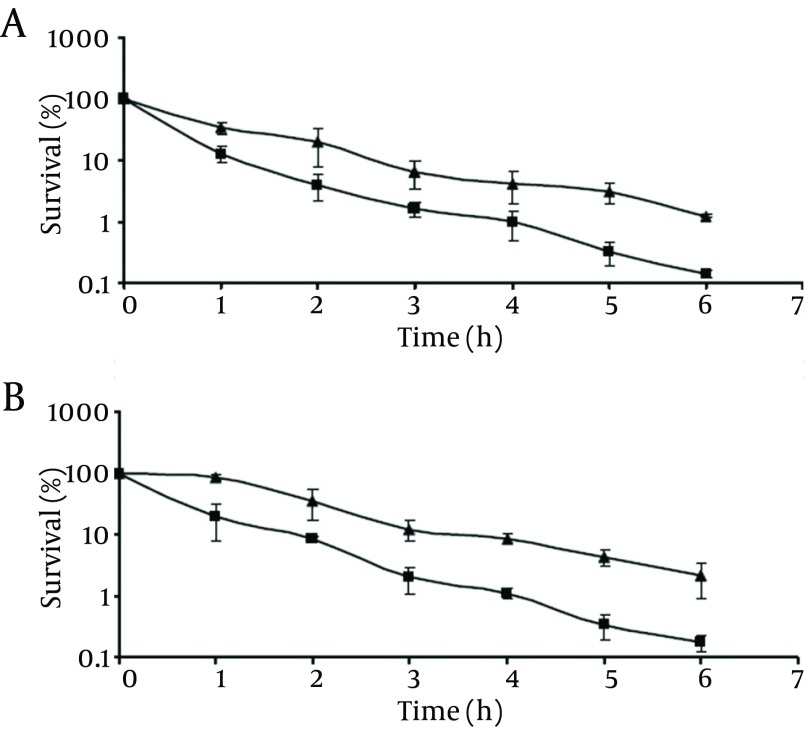
Survival of Pathogenic (A) and Non-Pathogenic (B) *V. parahaemolyticus* After Exposure to TSB Containing 20% NaCl: Control Cells (▲); Chlorine-Stressed Cells (■) The initial populations were 10^4^-10^5^cfu/mL. Survival rates were obtained by dividing the survival populations by the initial populations which corresponds to 100%.

As shown in Figure 5, the survival rate of the chlorine stressed cells significantly decreased (P < 0.05) compared to those of the control cells as the incubation time was extended. At the end of 6-hour incubation, control cells of pathogenic and non-pathogenic strains showed survival rates of 19.3% and 14.5%, respectively. Meanwhile, chlorine-stressed cells of both strains exhibited significantly (P < 0.05) lower survival rates of 5.6% and 4.0%, respectively. In addition, comparing the survival rates of chlorine-stressed cells of pathogenic and non-pathogenic *V. parahaemolyticus* showed no significant difference (P > 0.05), similar to the normal cells.

## 5. Discussion

 The present study first compared the survival of normal cells of pathogenic and non-pathogenic *V. parahaemolyticus* under adverse conditions. According to the obtained results, although the survival rates of pathogenic and non-pathogenic strains decreased as the incubation time at 50°C increased (Figure 1A), no significant difference was observed. Besides, viable population of pathogenic and non-pathogenic *V. parahaemolyticus* decreased (P > 0.05) at low temperatures, especially at -18°C (Figure 1B and 1C). This is thought to be due to the entrance of *V. parahaemolyticus* into a viable but nonculturable (VBNC) state. It has been previously demonstrated that this inability to culture certain *Vibrio* species from low-temperature environments is not due to the cell death, but to the viable but nonculturable state, which defines the inability of cells to produce colonies on appropriate solid media even following prolonged incubation (15).

To evaluate the effect of chlorine stress on the susceptibility of pathogenic and non-pathogenic *V. parahaemolyticus* to various environmental stresses, the current study demonstrated that chlorine stress made *V. parahaemolyticus* cells, more susceptible to incubation at 4°C, regardless of their pathogenicity, The higher susceptibility of chlorine-stressed cells to low storage temperature could be due to the injuries caused by chlorine in viable population of *V. parahaemolyticus*. When chlorine gas or aqueous solutions of calcium and sodium hypochlorite is added to water, a reaction occurs splitting it into hypochlorous acid and hypochlorite ions. The hypochlorous acid is the active killing form of chlorine. This chlorine molecule easily enters microorganisms through their cell walls and kills the organisms by destroying the sulfur groups on the cells’ enzymes, causing cells to stop metabolism, and resulting in the cell death. The amount of hypochlorous acid and hypochlorite ion produced following chlorine addition to water is directly related to the pH of the water. At a pH of 7.5 (the current study) the amount of hypochlorous acid produced was about 50% (3). Chlorine, at low level, is only lethal for a small number of exposed cells but induces injury in a large proportion of the remaining bacterial population (9). According to the previous reports, cold shock resulted in an enhanced resistance of *V. parahaemolyticus* (14) and ethanol shock induced no significant alteration in survival of *V. parahaemolyticus* at 4°C (13).

However, chlorine stress did not significantly alter the survival of pathogenic and non-pathogenic *V. parahaemolyticus* at -18°C. This may be due to the entrance of a large population of pathogenic and non-pathogenic *V. parahaemolyticus*, regardless of the chlorine stress, into a viable but nonculturable state following the incubation at freezing temperature. In addition, formation of ice crystals and solute concentration effect, which occurs at freezing temperature, are detrimental to microorganisms (16). Contrary to the results of the current study, enhanced susceptibility of *V. parahaemolyticus* at -18°C after ethanol shock treatment had been previously reported (13).

Although ethanol shock resulted in an enhanced resistance of *V. parahaemolyticus* (13), and cold shock made the cells more susceptible to high temperature (13), chlorine stress induced no significant alteration (P > 0.05) in the survival of pathogenic and non-pathogenic *V. parahaemolyticus* at 50°C. High concentrations of NaCl may lead to plasmolysis, injury and death of microorganisms (17). Therefore, in addition to impart salty flavor, NaCl is commonly used in various steps of food preparation to inhibit the growth of spoilage and pathogenic bacteria. It has been previously reported that environmental stress might affect the susceptibility of microorganism to high salt content. For example, *V. parahaemolyticus* became more susceptible to high NaCl content after exposure to ethanol shock (2) or heat shock treatments (18). In the present study, chlorine stress was also found to enhance the susceptibility of pathogenic and non-pathogenic *V. parahaemolyticus* to high NaCl (P < 0.05).

The results of the present study demonstrated that pathogenic and non-pathogenic *V. parahaemolyticus* had the same survival characteristics under adverse conditions. They also behaved similarly after chlorine stress. Moreover, chlorine stress increased the susceptibility of pathogenic and non-pathogenic *V. parahaemolyticus* to incubation at 4°C and the presence of high NaCl content in the medium. However, chlorine stress neither significantly alter the thermal tolerance of pathogenic and non-pathogenic *V. parahaemolyticus*, nor the susceptibility to freezing temperature. These results should be considered in the development of food preservation measures.
